# Relationship between serum gamma-glutamyl transferase level and colorectal adenoma

**DOI:** 10.1371/journal.pone.0240445

**Published:** 2020-10-13

**Authors:** Tzu-Chan Hong, Hung-Chih Yang, Chi-Ling Chen, Jia-Horng Kao, Chun-Jen Liu, Ming-Jen Chen, Horng-Yuan Wang, Yang-Che Kuo, Lo-Yip Yu, Kuang-Chun Hu

**Affiliations:** 1 Department of Internal Medicine, National Taiwan University Hospital, National Taiwan University College of Medicine, Taipei, Taiwan; 2 Graduate Institute of Clinical Medicine, College of Medicine, National Taiwan University, Taipei, Taiwan; 3 Division of Gastroenterology, Department of Internal Medicine, MacKay Memorial Hospital, Taipei, Taiwan; 4 Health Evaluation Center, MacKay Memorial Hospital, Taipei, Taiwan; 5 MacKay Junior College of Medicine, Nursing, and Management, Taipei, Taiwan; Nagoya University, JAPAN

## Abstract

**Background and aims:**

Cost-effective serology tests may increase the predictive accuracy of colonoscopy for colorectal cancer screening. Reportedly, gamma-glutamyl transferase (GGT) is associated with oxidative stress and carcinogenesis and has been found to be elevated in the serum of cancer patients and colorectal adenoma tissue. We aimed to investigate the association between serum GGT levels and colorectal adenoma.

**Methods:**

This single-center, health examination-based cohort enrolled 2475 subjects from 2006 to 2015. Baseline characteristics, laboratory data, bidirectional gastrointestinal endoscopy, and transabdominal ultrasonography were used to evaluate the severity of fatty liver.

**Results:**

We found an elevated median GGT level in subjects with tubular adenoma compared with those without (23 IU/L and 20 IU/L, p<0.001). A GGT cutoff of ≥20 IU/L reached a maximal Youden index in receiver operating curve (ROC) analyses. Subsequent regression analyses showed an odds ratio of 1.46 (95% CI 1.17–1.82, p<0.001) for age, body mass index, diabetes diagnosis, total cholesterol, triglycerides, low-density lipoprotein cholesterol, and positive Helicobacter pylori urease test, all being associated with an increased incidence of colon adenoma. Subgroup analysis showed that the odds ratio (OR 1.27, 95% CI 1.15–1.68, p<0.001) is only significant and highest in patients with a negative or mild fatty liver and an ALT level of ≤40 IU/L.

**Conclusions:**

The results suggested a positive correlation of GGT with colon adenoma incidence and a predictive value with a cutoff point of >20 IU/L, which is within the normal range. The effect may be most prominent for those without steatohepatitis.

## Introduction

Colon adenoma-carcinoma sequence has been known as a major developmental pathway for colon cancer since it was first published in 1978 [1] and confirmed in this era in which molecular biology and genetics define the pathogenesis of colon cancer [2]. Screening tests can detect early precancerous lesions and enable timely intervention, and specific screening tests for colorectal adenoma include colonoscopy, fecal immunochemical tests (FIT), fecal DNA tests, computed tomography colonography, capsule colonoscopy, and Septin-9 assay. However, apart from colonoscopy and FIT, no appropriate serology marker has proved to be a cost-effective screening for colorectal adenoma [3].

The development of precancerous lesions was proposed to be associated with oxidative stress [4]. The serum gamma-glutamyl-transferase (GGT) level is one of the biochemical tests that reflect cellular oxidative stress and are associated with the glutathione metabolism pathway by which cells manage pro-oxidants [4, 5]. It was also suggested that GGT was associated with inflammation by mitogen-activated protein kinase activation in response to oxidative stress, in addition to cytokines, including tumor necrosis factor, and interferon which also drive precancerous neoplasm formation [5]. In population-based studies, elevated GGT was found to predict cancer incidence in a dose-response relationship and was also associated with digestive organ malignancy in both men and women [6, 7].

The use of GGT in the detection of colonic neoplasms has not been fully established. A pilot study focusing on the imaging of colorectal neoplasms using a GGT-activatable fluorescent probe documented a promising tool for both endoscopic and pathologic evaluation [8]. However, no previous studies have addressed the relationship between the level of serum GGT and colonic polyps or adenoma. In this study, we investigated the association of serum GGT levels with colorectal adenoma, and its optimal cutoff point, as a possible future non-invasive screening tool.

## Materials and methods

### Patient population

This was a retrospective case-control study that included 4669 subjects who underwent health checkups at MacKay Memorial Hospital, Taipei, Taiwan, from January 2006 to December 2015. The inclusion criteria were: age over 40 years-old, patients who underwent esophagogastroduodenoscopy (EGD) with a urease test performed during the procedure and a colonoscopy on the same day. Patients were excluded for a proven history of acute myocardial infarction or stroke, high risk or proven colorectal cancer, an incomplete colonoscopy, or inadequate bowel preparation, lacking biopsy-based urease test for Helicobacter pylori by EGD. After exclusion, a total of 2475 patients were enrolled in the study. The institutional review board of the MacKay Memorial Hospital approved this study and waived the requirement for informed consent as we used only de-identified data collected as part of routine screening visits. (12MMHIS163).

### Demographic and clinical data collection

Baseline subject characteristics including age, gender, height, weight, personal medical history, family history, and smoking were obtained from a questionnaire administered on the day of examination (S1 and S2 Files). Blood pressure was checked and laboratory tests conducted. These included total cholesterol, triglycerides (TG), low-density lipoprotein (LDL), hemoglobin A1C (HbA_1c_), and Glucose *Ante Cibum* (AC). Individuals with type 2 diabetes mellitus (DM) were diagnosed with one of the following criteria: HbA1c≥6.5, or fasting glucose≥126 [9–11]. EGD was performed with gastro-fibro scope (GIF Q260; Olympus Optical, Tokyo, Japan) and rapid urease test (CLO, Pronto-Dry; Gastrex Corp., Warsaw, Poland) was performed on the gastric antral biopsy specimen. Colonoscopy was performed using the CF Q260AL colonoscope (Olympus Optical) by an experienced endoscopist. Colonic polyps were removed by biopsy forceps if smaller than 0.5 cm, and with a polypectomy snare if larger than 0.5 cm. Patients were classified into three groups based on colonic polyp findings: polyp-free, hyperplastic polyps, and adenomatous polyps. Those in which pathologic diagnosis showed inflammatory or juvenile polyps, lipomas, lymphoid aggregates, and chronic non-specific inflammation were classified in the polyp-free group. Transabdominal ultrasonography was also performed, and fatty liver was evaluated and classified into negative, mild, moderate, and severe. The definition of mild fatty liver is having slightly increased echogenicity of the liver parenchyma compared to the kidneys. For moderate fatty liver, moderately increased echogenicity of the liver was associated with blunting of the portal vein wall and slight attenuation of the diaphragm. Severe fatty liver required significantly increased echogenicity and posterior attenuation of the liver and diaphragm and the visualization of intrahepatic vessel walls and gallbladder walls were lost.

### Statistical analysis

Statistics were performed using NCSS 12 Statistical Software (NCSS, LLC. Kaysville, Utah, USA, 2018. ncss.com/software/ncss). Continuous demographic data were described using median and IQR and were compared using the non-parametric Wilcoxon Rank-Sum tests as some of the laboratory data did not follow a normal distribution. Categorical data were compared using Chi-squared analyses. The effects of different parameters on adenoma incidence were analyzed using a logistic regression model. Univariable analyses were performed first and those variables with p<0.05 were used in multivariate logistic regression. Statistical significance was defined as p<0.05 in this study.

## Results

### Demographic data for colorectal adenoma patients

The demographics for patients with tubular adenoma (n = 693) and those without (n = 1783) in this health examination-based cohort are listed and compared in Table 1. The population with tubular adenoma has an older median age, higher proportion of male gender, slight, but significant, increase in BMI, higher proportion of smoking, diagnosis of DM, and higher median TG, ALT, and GGT, and proportion of positive CLO test by EGD and fatty liver by abdominal ultrasonography.

**Table 1:** Demographics of participants with and without colon tubular adenoma.

	Tubular Adenoma (-)	Tubular Adenoma (+)	P value
	n = 1783	n = 692	
Median Age (IQR)	51.50 (14.48)	56.71 (12.36)	<0.001
Gender male	64.25%	73.84%	<0.001
Median BMI (IQR)	24.1 (4.20)	24.9 (4.5)	<0.001
Smoking	22.50%	27.60%	<0.01
DM diagnosis	9.87%	19.22%	<0.001
Median Total Cholesterol (IQR)	202 (50)	200 (49)	0.62
Median TG (IQR)	117 (83.5)	128 (87.75)	<0.001
Median LDL (IQR)	134.5 (48)	135 (49.75)	0.60
Median ALT (IQR)	22 (8)	23 (9)	0.017
Median GGT (IQR)	20 (17)	23 (17)	<0.001
CLO test	37.30%	46.10%	<0.001
Fatty liver	57.31%	65.87%	<0.001

Demographic data and distribution of data set was listed. Units for biochemical values are as following: Total cholesterol (mg/dL), TG (mg/dL), LDL (mg/dL), ALT (U/L), GGT (U/L).

### The optimal cutoff point of GGT in the prediction of colorectal adenoma

**Fig 1:**
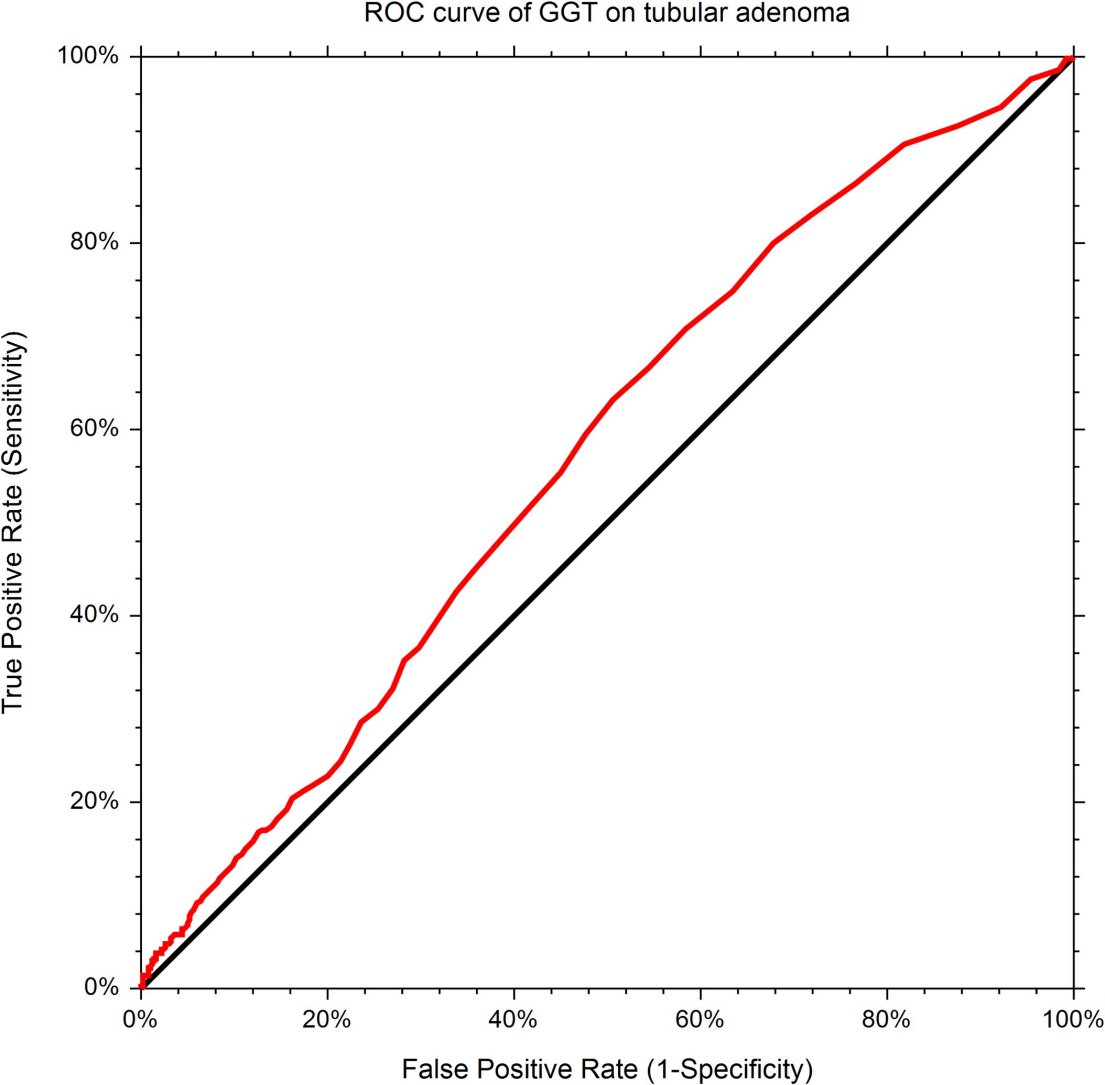
ROC curve of GGT on the prediction of tubular adenoma incidence.

Due to the higher GGT in adenoma-positive patients, ROC curve and area under the curve (AUC) analysis were performed in the study population. The AUC of GGT to predict colorectal adenoma is 0.57 with a 95% CI of AUC 0.55–0.60; while the highest Youden index (1.125) falls on the cutoff value of ≥20 U/L. The sensitivity at this cutoff point is 0.63 while the specificity is 0.49 (Fig 1).

### Regression analyses for colorectal adenoma risk factors

Univariate regression analysis was performed first. All the variables chosen for study revealed a statistically significant association with the incidence of colorectal adenoma. Multivariate logistic regression analysis was then applied to the variables and showed that age (OR 2.65, 95% CI 2.42–2.89, p<0.001), BMI (OR 0.94, 95% CI 0.92–0.96, p<0.001), diagnosis of DM (OR 1.52, 95% CI 1.15–1.99, p<0.001), total cholesterol (OR 0.97, 95% CI 0.97–0.98, p<0.001), TG (OR 1.003, 95% CI 1.002–1.005, p<0.001), LDL (OR 1.02, 95% CI 1.01–1.03, p<0.001), GGT≥20 U/L (OR 1.46, 95% CI 1.17–1.82, p<0.001) and CLO test (OR 1.39, 95% CI 1.15–1.68, p<0.001) were associated with an increased risk of colorectal adenoma. However, male gender, smoking, ALT level, presence of fatty liver showed no statistical significance in the multivariate analysis (Table 2).

**Table 2:** Univariate and multivariate analyses.

	Univariate analysis			Multivariate analysis		
	OR	95% CI of OR	P value	OR	95% CI of OR	P value
Age	2.53	2.32–2.77	<0.001	2.65	2.42–2.89	**<0.001**
Gender male	1.15	1.09–1.22	<0.001	1.00	0.79–1.26	0.99
BMI	2.52	2.31–2.77	<0.001	0.94	0.92–0.96	**<0.001**
Smoking	1.23	1.06–1.42	<0.001	1.15	0.91–1.45	0.24
DM diagnosis	1.95	1.58–2.40	<0.001	1.52	1.15–1.99	**<0.001**
Total cholesterol	2.56	2.34–2.79	<0.001	0.97	0.97–0.98	**<0.001**
TG	2.56	2.34–2.79	<0.001	1.003	1.002–1.005	**<0.001**
LDL	2.56	2.35–2.80	<0.001	1.02	1.01–1.03	**<0.001**
ALT	2.48	2.27–2.71	<0.001	1.00	0.99–1.00	0.67
GGT ≧ 20	1.25	1.16–1.34	<0.001	1.46	1.17–1.82	**<0.001**
CLO test	1.24	1.12–1.37	<0.001	1.39	1.15–1.68	**<0.001**
Fatty liver	1.15	1.07–1.23	<0.001	1.09	0.87–1.37	0.46

Logistic regression was used for odds ratios among parameters contributing to the incidence of tubular adenoma. Units for biochemical values are as following: Total cholesterol (mg/dL), TG (mg/dL), LDL (mg/dL), ALT (U/L), GGT (U/L).

### The influence of fatty liver and steatohepatitis on GGT in prediction of colorectal adenoma

To further clarify the relationship between fatty liver, steatohepatitis, and the incidence of colorectal adenoma, subgroup analyses based on these factors were performed. Fatty liver severity was classified into two groups, including negative or mild and moderate or severe, based on the abdominal ultrasonography. The odds ratio of GGT≥20 U/L for the colorectal adenoma incidence is only statistically significant in the population with negative or mild fatty liver (OR 1.27, 95% CI 1.14–1.41, p<0.001) but not with moderate or severe fatty liver (OR 1.07, 95% CI 0.99–1.17, p = 0.1) (Table 3). Moreover, those with negative or mild fatty liver with an ALT≤40 is the only statistically significant subgroup (OR 1.32, 95% CI 1.16–1.50, p<0.001) when analyzing the severity of fatty liver and liver function tests (Table 3).

**Table 3:** (a) Odds ratio of GGT on adenoma incidence in different groups of fatty liver severity. (b) Odds ratio of GGT on adenoma incidence in different groups of fatty liver and hepatitis severity.

(a) Odds ratio of GGT on adenoma incidence in different groups of fatty liver severity^a^				
		OR	95% CI	P value
**Fatty liver negative or mild**				
	GGT<20	1		
	GGT≧20	1.27	1.14–1.41	<0.001
**Fatty liver moderate or severe**				
	GGT<20	1		
	GGT≧20	1.07	0.99–1.17	0.10
(b) Odds ratio of GGT on adenoma incidence in different groups of fatty liver and hepatitis severity^b^				
		OR	95% CI	P value
**Fatty liver negative or mild ALT**≦**40**				
	GGT<20	1		
	GGT≧20	1.32	1.16–1.50	<0.001
**Fatty liver negative or mild ALT>40**				
	GGT<20	1		
	GGT≧20	1.04	0.95–1.15	0.37
**Fatty liver moderate or severe ALT**≦**40**				
	GGT<20	1		
	GGT≧20	1.12	0.99–1.28	0.08
**Fatty liver moderate or severe ALT>40**				
	GGT<20	1		
	GGT≧20	1.05	0.97–1.13	0.25

^a^Units for biochemical values are as following: GGT(U/L).

^b^Units for biochemical values are as following: ALT(U/L), GGT(U/L).

## Discussion

In this study, we proposed the novel use of an old biomarker, serum GGT level, for colorectal adenoma risk assessment. The finding was generated from a validated health examination cohort. Previously identified risk factors including age, diagnosis of DM, TG, LDL, and *H*. *pylori* infection were reproducibly detected in this study [10, 11]. We discovered a higher median level of GGT in subjects with colorectal adenoma than those without. A lower cutoff point (20 IU/L) than the upper limit in a normal population was determined by the ROC curve for colorectal adenoma prediction. Multivariate logistic regression analyses also revealed a significant odds ratio of 1.48 for GGT≥20 IU/L on the incidence of colorectal adenoma. This finding, in the subsequent subgroup analyses, is only true and significant in those with negative or mild fatty liver without elevated liver function tests.

GGT activity has been found to be elevated in the process of colon carcinogenesis in colonic tissue both in experimental animal models [12, 13] and in humans [8]. Serum GGT, however, has not been fully elucidated in the process of colon adenoma-carcinoma sequence. Our study provides the first evidence of elevated serum GGT level, from a normal level, associated with colorectal adenoma incidence. In some earlier studies, a similar association had been noted; higher levels of GGT, even though within normal limits, were found to be associated with an increased risk of neoplasia. In two population-based studies, the VHM&PP Study group in Austria revealed an increased overall cancer risk associated with mildly elevated GGT (≥36 U/L) in females. A positive dose-response relationship of GGT levels has also been associated with an increased relative risk for all cancers including digestive organ malignancies when compared to a set baseline of GGT = 25 U/L in male populations. [6, 7]. The AMORIS study in Sweden also showed the dose-response relationship of overall cancer or digestive cancer risk from high-normal levels of GGT (≥18 U/L). The results also indicated a more significant association between GGT and cancer in individuals with lower levels of GGT (≥18 IU/L) and nearly normal liver function tests (ALT <50 IU/L) [14].

The results of our study provide evidence that the elevation of GGT may be also associated with adenoma of the colon, a pre-malignant lesion of colon cancer. The cutoff level is also at the higher end of the normal range. We also found that the association between elevated GGT and the risk of colorectal adenoma was more significant in those without steatohepatitis. Patients with alcoholic or non-alcoholic steatohepatitis (NASH) may also have elevated GGT. Only in subjects without a fatty liver, or with mild fatty liver and normal ALT level, might the GGT level be representative of body oxidative stress, which contributes to the process of carcinogenesis [15].

Our study, however, cannot conclude that there is a causal relationship between elevated GGT and the risk of colorectal adenoma because of the limitation of the cross-sectional cohort design. Previous prospective cohorts, however, are also not able to conclusively answer this question. We believe the role of elevated GGT is closer to that of a bystander in oxidative stress rather than a direct cause of neoplasms owing to its role in pre-malignant colonic lesions and the relationship with steatohepatitis in subgroup analyses. Our finding, however, may be useful in developing a future colorectal adenoma-carcinoma scoring system. It may be of use in countries with limited colonoscopic resources and it may be useful in combination with the FIT as a screening tool, adding more information that can improve the predictive accuracy and is worth studying in the future. In our analyses, other known tumor markers, especially carcinoembryonic antigen (CEA), which is well studied in cancerous conditions but lacks solid evidence for its correlation with colorectal adenoma, were not as predictive as GGT in terms of AUC and odds ratio in the regression, considering smoking status.

In conclusion, our study described the first positive association between serum GGT levels and colorectal adenoma incidence. The cutoff point is in the normal range of GGT. Patients with minimal fatty liver and normal ALT levels have the strongest correlation between elevated GGT and pre-malignant colorectal lesions.

## Supporting information

S1 File The questionnaire administered to patients on the day of their examination (Mandarin). (DOC)

S2 File The questionnaire administered to patients on the day of their examination (English). (DOCX)
