# Supplementary material for: Relationship between serum gamma-glutamyl transferase level and colorectal adenoma
Source: PLoS One. 2020 Oct 13;15(10):e0240445. doi: 10.1371/journal.pone.0240445 (PMC7553303; doi:10.1371/journal.pone.0240445)
Supplement: S1 File — (DOC) [file pone.0240445.s001.doc]

**馬偕健檢中心健康調查問卷**

**Barcode 黏貼處**

*12345678*

12345678

您好平安，感謝您對馬偕健檢中心的支持與信賴。本中心特別製作健康問卷，請您仔細填寫，以便醫師瞭解並掌握您的健康狀況。^6^

**第一部分：基本資料**

1. **姓 名：** 檢查日：民國 年 月 日
2. **身分證號：** 生日**：**民國 年 月 日
3. **性 別：** ○ 男 ○ 女
4. **電 話：**手機：

日：( ) 夜：( )

1. **報告掛號地址：**□ □ □
2. **電子報告：**□ 不需 □ 需( E-mail： )
3. **婚姻狀況：** ○ 未婚 ○ 已婚 ○ 離婚 ○ 喪偶

**畫記說明：為了電腦作業順利，煩請您務必在選項的**○**內打**●

**第二部分：生活習慣與飲食**

1. **一般而言，您目前的健康狀況是：**

○ 非常好 ○ 好 ○ 普通 ○ 不好

1. **跟一年前比較，您的健康狀況是：**

○ 變好 ○ 不變 ○ 變差 ○ 沒感覺

1. **您一天都有吃兩種水果和三種蔬菜：**

○ 沒有 ○ 偶爾 ○ 經常 ○ 每日

1. **您三餐的主食有1~2餐選用全穀麥片：**

○ 沒有 ○ 偶爾 ○ 經常 ○ 每日

1. **您是否為素食者：**

○ 否 ○ 是 ○ 其他：

1. **您最近三個月體重增或減5%以上：**

○ 否 ○ 是 ○ 不知道

1. **您有無抽菸的習慣：**

○ 無 ○ 有 ○ 以前抽，現在已戒

1. **您有無喝酒的習慣：**

○ 無 ○ 有 ○ 以前喝，現在已戒

1. **您有無嚼檳榔的習慣：**

○ 無 ○ 有 ○ 以前嚼，現在已戒

1. **您每週是否至少運動3次，每次30分鐘？**

○ 否 ○ 是 ○ 其他：

**第三部分：個人及家族病史**

1. **您長期（每日）服用的藥物（可複選）：**

○ 無服用任何藥物 ○ 鎮定劑或安眠藥 ○尿酸藥物 ○ 類固醇藥物 ○ 心臟病藥物 ○ 荷爾蒙 ○ 高血壓 ○ 止痛藥 ○ 糖尿病藥物

○ 甲狀腺藥物 ○ 高血脂藥物 ○ 腸胃藥 ○ 中藥 ○ 精神科藥物 ○ 鐵劑 ○ 抗凝血劑 ○ 干擾素

○ 其他：_______________

1. **您有過敏史嗎（包括藥物、食物及其他，可複選）？**

○ 否 ○ 藥物**：**

○ 食物**：** ○ 其他**：**

1. **您曾罹患以下疾病或開刀過嗎？家族病史為何？（可複選）**

| **疾病** | **本人病史** | | **家族病史** | | | |
| --- | --- | --- | --- | --- | --- | --- |
|  | **罹患** | **開刀** | **父親** | **母親** | **子女** | **祖父母** |
| **高血壓** | ○ | ○ | ○ | ○ | ○ | ○ |
| **糖尿病** | ○ | ○ | ○ | ○ | ○ | ○ |
| **夜盲症** | ○ | ○ | ○ | ○ | ○ | ○ |
| **乾眼症** | ○ | ○ | ○ | ○ | ○ | ○ |
| **青光眼** | ○ | ○ | ○ | ○ | ○ | ○ |
| **白內障** | ○ | ○ | ○ | ○ | ○ | ○ |
| **高血脂症** | ○ | ○ | ○ | ○ | ○ | ○ |
| **心肌梗塞** | ○ | ○ | ○ | ○ | ○ | ○ |
| **心血管疾病** | ○ | ○ | ○ | ○ | ○ | ○ |
| **心衰竭** | ○ | ○ | ○ | ○ | ○ | ○ |
| **冠心病** | ○ | ○ | ○ | ○ | ○ | ○ |
| **腦中風** | ○ | ○ | ○ | ○ | ○ | ○ |
| **痛風** | ○ | ○ | ○ | ○ | ○ | ○ |
| **高尿酸症** | ○ | ○ | ○ | ○ | ○ | ○ |
| **脂肪肝** | ○ | ○ | ○ | ○ | ○ | ○ |
| **B型肝炎帶原** | ○ | ○ | ○ | ○ | ○ | ○ |
| **C型肝炎帶原** | ○ | ○ | ○ | ○ | ○ | ○ |
| **肝硬化** | ○ | ○ | ○ | ○ | ○ | ○ |
| **逆流性食道炎** | ○ | ○ | ○ | ○ | ○ | ○ |
| **消化性潰瘍** | ○ | ○ | ○ | ○ | ○ | ○ |
| **慢性胰臟炎** | ○ | ○ | ○ | ○ | ○ | ○ |
| **泌尿道結石** | ○ | ○ | ○ | ○ | ○ | ○ |
| **肺氣腫** | ○ | ○ | ○ | ○ | ○ | ○ |
| **膽囊/膽管結石** | ○ | ○ | ○ | ○ | ○ | ○ |
| **疾病** | **本人病史** | | **家族病史** | | | |
|  | **罹患** | **開刀** | **父親** | **母親** | **子女** | **祖父母** |
| **氣喘** | ○ | ○ | ○ | ○ | ○ | ○ |
| **紅斑性狼瘡** | ○ | ○ | ○ | ○ | ○ | ○ |
| **風濕性關節炎** | ○ | ○ | ○ | ○ | ○ | ○ |
| **甲狀腺疾病** | ○ | ○ | ○ | ○ | ○ | ○ |
| **貧血** | ○ | ○ | ○ | ○ | ○ | ○ |
| **地中海型貧血** | ○ | ○ | ○ | ○ | ○ | ○ |
| **結核性疾病** | ○ | ○ | ○ | ○ | ○ | ○ |
| **腎臟病** | ○ | ○ | ○ | ○ | ○ | ○ |
| **腎結石** | ○ | ○ | ○ | ○ | ○ | ○ |
| **攝護腺肥大** | ○ | ○ | ○ | ○ | ○ | ○ |
| **鼻咽癌** | ○ | ○ | ○ | ○ | ○ | ○ |
| **口腔癌** | ○ | ○ | ○ | ○ | ○ | ○ |
| **肺癌** | ○ | ○ | ○ | ○ | ○ | ○ |
| **乳癌** | ○ | ○ | ○ | ○ | ○ | ○ |
| **食道癌** | ○ | ○ | ○ | ○ | ○ | ○ |
| **胃癌** | ○ | ○ | ○ | ○ | ○ | ○ |
| **肝癌** | ○ | ○ | ○ | ○ | ○ | ○ |
| **胰臟癌** | ○ | ○ | ○ | ○ | ○ | ○ |
| **大腸直腸癌** | ○ | ○ | ○ | ○ | ○ | ○ |
| **卵巢癌** | ○ | ○ | ○ | ○ | ○ | ○ |
| **子宮頸癌** | ○ | ○ | ○ | ○ | ○ | ○ |
| **攝護腺癌** | ○ | ○ | ○ | ○ | ○ | ○ |
| **白血病** | ○ | ○ | ○ | ○ | ○ | ○ |
| **淋巴癌** | ○ | ○ | ○ | ○ | ○ | ○ |
| **其他：_______** | ○ | ○ | ○ | ○ | ○ | ○ |

**第四部分：全身系統症狀**（請依您最近一個月實際情形畫記）

選項說明：「不會」表示從不或很少發生此狀況

「偶爾」表示一個月內發生1~3次

「經常」表示一星期內發生1~3次

「每日」表示天天發生此狀況

| **全身系統症狀** | **不會** | **偶爾** | **經常** | **每日** |
| --- | --- | --- | --- | --- |
| 1. **您痰或鼻涕中有血絲嗎？** | ○ | ○ | ○ | ○ |
| 1. **您最近會持續咳嗽嗎？** | ○ | ○ | ○ | ○ |
| 1. **您有打噴嚏嗎？** | ○ | ○ | ○ | ○ |
| 1. **您有鼻塞嗎？** | ○ | ○ | ○ | ○ |
| 1. **您有流鼻血嗎？** | ○ | ○ | ○ | ○ |
| 1. **您有聲音變沙啞嗎？** | ○ | ○ | ○ | ○ |
| **全身系統症狀** | **不會** | **偶爾** | **經常** | **每日** |
| 1. **您有耳鳴嗎？** | ○ | ○ | ○ | ○ |
| 1. **您有吞嚥困難嗎？** | ○ | ○ | ○ | ○ |
| 1. **您有暈眩嗎？** | ○ | ○ | ○ | ○ |
| 1. **您有聽力困難嗎？** | ○ | ○ | ○ | ○ |
| 1. **您有牙齦出血的現象嗎？** | ○ | ○ | ○ | ○ |
| 1. **您平常咀嚼是否覺得牙齒疼痛？** | ○ | ○ | ○ | ○ |
| 1. **您張口或吃東西時是否會覺得臉部肌肉或關節疼痛嗎？** | ○ | ○ | ○ | ○ |
| 1. **您呼吸時聽到胸部有咻咻的聲音** | ○ | ○ | ○ | ○ |
| 1. **您近來有不尋常的食慾不佳嗎？** | ○ | ○ | ○ | ○ |
| 1. **您最近有不尋常的倦怠疲勞感嗎？** | ○ | ○ | ○ | ○ |
| 1. **您會覺得胸口有灼熱感嗎？** | ○ | ○ | ○ | ○ |
| 1. **您會有酸水向上衝到口中嗎？** | ○ | ○ | ○ | ○ |
| 1. **您肚子餓時，胃會痛嗎？** | ○ | ○ | ○ | ○ |
| 1. **您吃完飯後，胃會痛嗎？** | ○ | ○ | ○ | ○ |
| 1. **您用餐時很早就有飽足感嗎？** | ○ | ○ | ○ | ○ |
| 1. **您用餐後會腹脹不舒服嗎？** | ○ | ○ | ○ | ○ |
| 1. **您吞嚥時食物易卡在喉嚨或胸部** | ○ | ○ | ○ | ○ |
| 1. **您最近排便有費力情形嗎？** | ○ | ○ | ○ | ○ |
| 1. **您最近糞排呈團塊或硬結外型嗎？** | ○ | ○ | ○ | ○ |
| 1. **您最近排便有解不淨的感覺嗎？** | ○ | ○ | ○ | ○ |
| 1. **您最近排便時有肛門阻塞感嗎？** | ○ | ○ | ○ | ○ |
| 1. **您最近大便帶有鮮血嗎？** | ○ | ○ | ○ | ○ |
| 1. **您最近大便有黑便現象嗎？** | ○ | ○ | ○ | ○ |
| 1. **您下肢有浮腫現象嗎？** | ○ | ○ | ○ | ○ |
| 1. **您的關節有紅腫變形嗎？** | ○ | ○ | ○ | ○ |
| 1. **您會腰酸背痛**（非運動後）**嗎？** | ○ | ○ | ○ | ○ |
| 1. **您有漏尿的情形嗎？** | ○ | ○ | ○ | ○ |
| 1. **您小便要等很久才尿出來嗎？** | ○ | ○ | ○ | ○ |
| 1. **您小便時，有痛或灼熱的感覺嗎？** | ○ | ○ | ○ | ○ |
| 1. **您近來有尿中帶血的情形嗎？** | ○ | ○ | ○ | ○ |
| 1. **您有急尿感難以忍住嗎？** | ○ | ○ | ○ | ○ |
| 1. **您排尿次數增加**（一天超過八次） | ○ | ○ | ○ | ○ |
| 1. **您小便完畢後感覺仍未完全排空** | ○ | ○ | ○ | ○ |
| **全身系統症狀** | **不會** | **偶爾** | **經常** | **每日** |
| 1. **您小便時需要用力或常會中斷，須分次尿完嗎？** | ○ | ○ | ○ | ○ |
| 1. **我感覺緊張不安** | ○ | ○ | ○ | ○ |
| 1. **我覺得容易苦惱或動怒** | ○ | ○ | ○ | ○ |
| 1. **我對任何事都不感興趣** | ○ | ○ | ○ | ○ |
| 1. **我感覺憂鬱、心情低落** | ○ | ○ | ○ | ○ |
| 1. **我覺得比不上別人** | ○ | ○ | ○ | ○ |
| 1. **我有自殺的想法** | ○ | ○ | ○ | ○ |
| 1. **您看遠方視力會模糊嗎？** | ○ 否 ○ 是 | | | |
| 1. **您眼睛覺得紅癢乾澀嗎？** | ○ 否 ○ 是 | | | |
| 1. **您有飛蚊症症狀嗎？** | ○ 否 ○ 是 | | | |
| 1. **您閱讀時不戴眼鏡看會覺得模糊** | ○ 否 ○ 是 | | | |
| 1. **您曾有過短暫失去記憶嗎？** | ○ 否 ○ 是 | | | |
| 1. **您頸部有硬塊嗎？** | ○ 否 ○ 是 | | | |
| 1. **您有牙齒敏感的問題嗎？** | ○ 否 ○ 是 | | | |
| 1. **您有每半年定期做牙科檢查嗎？** | ○ 否 ○ 是 | | | |
| 1. **您口中是否有潰爛或白點存在？** | ○ 否 ○ 是 | | | |
| 1. **您身體表面有久不痊癒的傷口？** | ○ 否 ○ 是 | | | |
| 1. **您痣的顏色或大小是否有變化？** | ○ 否 ○ 是 | | | |
| 1. **您每周排便次數小於三次嗎？** | ○ 否 ○ 是 | | | |
| 1. **您一天排3次以上水便或不成形便嗎？**（腹瀉） | ○ 否 ○ 是 | | | |
| 1. **您是否患有痔瘡？** | ○ 否 ○ 是 | | | |
| 1. （81-88女性作答）**您目前是否懷孕** | ○ 否 ○ 是 | | | |
| 1. **您乳頭是否有疼痛或硬塊現象**（與經期無關的）**？** | ○ 否 ○ 是 | | | |
| 1. **您乳頭是否有分泌物或乳部變形現象？** | ○ 否 ○ 是 | | | |
| 1. **您月經週期是否不規則？** | ○ 否 ○ 是 | | | |
| 1. **您是否有經期外出血的情形？** | ○ 否 ○ 是 | | | |
| 1. **您月經量相較過去有顯著增加嗎？** | ○ 否 ○ 是 | | | |
| 1. **您性行為後是否有出血情形？** | ○ 否 ○ 是 | | | |
| 1. **您是否已經停經？** | ○ 否 ○ 是 | | | |
| 1. （89-90男性作答）**您兩側睪丸大小差異很大？** | ○ 否 ○ 是 | | | |
| 1. **您陰莖有反覆疼痛的情形嗎？** | ○ 否 ○ 是 | | | |

**第五部分：睡眠評估**（請依您最近一個月實際情形畫記）

1. **您每天睡眠時間平均為多少小時：**

○ 0~4 ○ 4~6 ○ 6~8 ○ > 8 小時

1. **您有日間昏昏欲睡**（嗜睡）**的情形：**

○ 沒有 ○ 偶爾 ○ 經常 ○ 每日

1. **您覺得自己的睡眠品質如何？**

○ 非常好 ○ 好 ○ 不好 ○ 很差

1. **您有無下列夜眠干擾情形（可複選）：**

○ 30分鐘內無法入眠 ○ 能入睡但易被吵醒

○ 夜間起來排尿2次以上 ○ 半夜或清晨便清醒

○ 有疼痛無法入睡 ○ 覺得呼吸不順暢

○ 作惡夢或多夢 ○ 大聲打鼾或咳嗽

○ 須用藥物助眠 ○ 以上均無

**第六部分：心肺功能與心絞痛/胸悶指數評估**

1. **以下心肺功能的自我評估，您覺得自己的狀態是：**

○ 一般活動不會喘

○ 一般活動輕度喘，休息或適度運動就感覺舒適

○ 一般活動輕度喘，只有休息感覺舒適

○ 任何活動均不舒適，休息時也會喘

1. **以下心絞痛 / 胸悶指數的功能分類，您覺得自己的狀態是：**

○ 一般活動不會胸悶

○ 迅速爬樓梯或情緒有壓力或走超過2棟建築物區塊會胸悶

○ 正常步調走路約1-2棟建築物區塊會胸悶

○ 休息時會發生胸悶

**第七部分：其他資料**

1. 服務機關**：** 職稱**：**
2. 教育程度**：** ○ 高中(職)以下 ○ 專科

○ 大學 ○ 研究所以上

1. 個人年所得**：** ○ 無 ○ < 60萬 ○ 60~100萬

○ 100~160萬 ○ 160萬以上

1. 工作場所中有無危害健康的因素**：**

○ 無 ○ 有：

1. 本次健康檢查的目的**：**

○ 定期檢查 ○ 公司安排 ○ 公職健檢 ○ 身體不適： ○ 其他：

1. 近半年是否有至門診看診？

○ 沒有

○ 有… ○ 馬偕 ○ 他院

看診次數 ○ 1~3次 ○ 4~6次 ○ 7次以上

感謝您的作答 馬偕健康檢查中心關心您的健康
