# Supplementary material for: Relationship between serum gamma-glutamyl transferase level and colorectal adenoma
Source: PLoS One. 2020 Oct 13;15(10):e0240445. doi: 10.1371/journal.pone.0240445 (PMC7553303; doi:10.1371/journal.pone.0240445)
Supplement: S2 File — (DOCX) [file pone.0240445.s002.docx]

Mr./Ms.

Thank you for choosing our health services. Before the health check there are some matters to remind you, and inform the process. Please take the time to read it, and prepare the required items to our center on time.

Your examination date： Y M D

**Registration Time：□ 7:40 □ 8:00 □ 8:00~9:00 □ 9:00~9:30 □**

Registration Place：16F., No.92, Sec. 2, Zhongshan N. Rd., Zhongshan Dist., Taipei City (Shuanglian station)

Direction：Please take the elevator #18~20 in front of the outpatient injection room, directly to the 16^th^ floor Health Evaluation Center.。

**【Health Checks Notification】**

| Before the Health Check ~ |
| --- |
| 1. Please eat **light diet** three days before the health check and avoid socializing, eat too much and drink alcohol. 2. **Please collect the stool sample 1 to 2 days before the health check (before drinking bowel cleaning liquid) and stored in a dry place.** 3. **Please fill out the health questionnaire and all kinds of consent and give to the staff when you arrive.** 4. Please do not eat or drink anything including water, chewing gum, or smoking **after 12 pm (fasting 8 hours**) before the health check day. 5. **Please bring your prescription drugs such as hypertension, diabetes, heart disease…etc,** after finish the fasting examinations, then can prescribe the drugs. 6. **Do not bring valuables belongings. Do not wear underwear which have jewellery, zippers and buttons.** 7. If you have eye exams and painless anesthesia exams, **do not wear contact lenses and do not drive the car. (The hospital parking outsourcing, the cost is 60NT/hr;We do not provide free parking.)** 8. For your convenience, you can use a credit card. **(only VISA and Master)** 9. We provide personal locker, sportswear, slippers and meals.   **◎Female Notification：**   1. Please avoid the **menstrual period for the health examination.**。 2. **If you suspect or already pregnant will not suitable for health check.**。 3. Please **do not collect stool sample, urine sample and pap smear** during menstrual period.。 4. **Three days before the pap smear**, **please do not**…   **I Intravaginal cleaning II. Use the drugs in the vaginal**  **III. Tub bath IV. Sexual behavior** |

**【Attention】**

| Gastroscopy |
| --- |
| 1. If you have **Glaucoma, asthma, heart disease or drugs allergy,** please inform the medical staff. 2. Do not use the **lipstick and nail polish**. |

| Colonscopy |
| --- |
| 1. **Please eat a low-residue diet and drink more water two days before the test**，should not eat any fried, high-fiber, high-protein foods.(vegetables, fruits, dairy product) 2. **If you have constipation habit, please take a week earlier to eat low-residue diet.** 3. **Please eat liquid diet the day before the examination. (fish soup, porridge, residues juice)** 4. **Please be sure to control low-residue diet. If the bowel is not cleaning good enough and need to rescheduled the test, we will have to charge 500NT.** |

| Painless anesthesia |
| --- |
| **The following conditions are high risks and not recommended for anesthesia:**   1. **Older than 75 years old.** 2. **Body Mass Index(BMI)＞35 (BMI = Weight (Kg) / (Height X Height) (M)** 3. Unstable angina, severe arrhythmia and who has myocardial infraction within six months. 4. Severe chronic lung disease or in acute status, Respiratory tract structural abnormal. 5. Had a stroke in previous one month. Used to have allergy to narcotic drugs. 6. Other diseases dependent on doctors assess. |

| Brain wave and MRI |
| --- |
| 1. Please wash your hair before the examination and **do not smear hair oil or hail gel.** 2. If you have implant equipment or other metal objects please inform us. **Cardiac pacemakers or cochlear implant should not do the MRI**. |

| Carrying Items |
| --- |
| □ 1.Stool container □ 3.Health insurance card, ID card, credit card。  □ 2.Questionnaire, consents □ 4. Prescription drugs |

**【Health check process】(for reference only）**

| Time | Process |
| --- | --- |
| Morning | Register、pay the bill、Questionnaire、Consents、Stool sample |
|  | Changing clothes、Blood test、Urine test、Height、Weight、Body fat |
|  | Abdominal echo、breast echo、MRI、CT |
|  | X-ray（Chest、Abdominal、Cervical、Lumbar、Full mouth） |
|  | Resting EKG、Hearing test、Gastroscopy |
|  | Vision、Intraocular pressure、Lung function(Male） |
|  | Colonoscopy、Carotid echo |
|  | Mammography、Echocardiography、Bone density |
|  | Brunch |
| Afternoon | Ophthalmology、Dental、ENT、Brain wave |
|  | Urology（Male）、Prostate echo、Kidney echo |
|  | Obstetrics&Gynecology（Female）、Pap smear、Gynecological echo |
|  | Physical exam、Report commentary、Tea time |
|  | Exercise EKG |
| 16:30 | End |

**【Postponement】** For rescheduled, please call us three days before the examination or 500NT will be charged.
**【Report】**

**After all the tests are completed in two weeks, we will send the report by registered and you may get the report about three weeks.** If you need electronic files please be informed us in advance.。

**The mailing address is limited in Taiwan** (including Kinmen, Matsu and Penghu). To send abroad, please inform us and we need to charge extra 500NT.。

Service phone：02-2511-8085

**Health Examination Notified**

**And**

**Process Description**


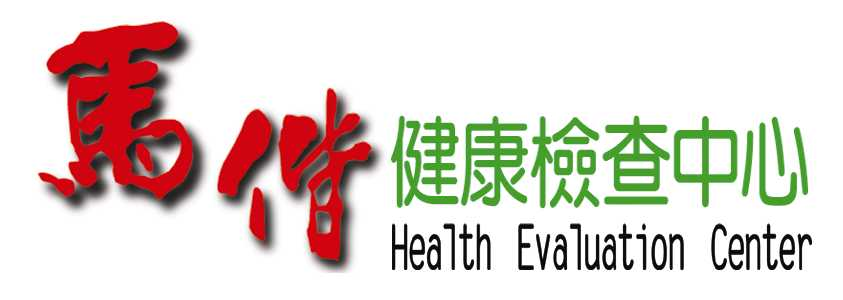


My dear brother, I wish you all flourished and healthy, just as your soul flourished **(聖經，約翰三書第二節)**
